# Validation of Susceptibility Loci for Vitiligo Identified by GWAS in the Chinese Han Population

**DOI:** 10.3389/fgene.2020.542275

**Published:** 2020-12-03

**Authors:** Lu Cheng, Bo Liang, Xian-Fa Tang, Xin-Ying Cai, Hui Cheng, Xiao-Dong Zheng, Jie Zheng, Meng-Wei Wang, Jun Zhu, Fu-Sheng Zhou, Pan Li, Feng-Li Xiao

**Affiliations:** ^1^Department of Dermatology of First Affiliated Hospital, Institute of Dermatology, Anhui Medical University, Hefei, China; ^2^Key Laboratory of Dermatology, Anhui Medical University, Ministry of Education, Hefei, China; ^3^The Center for Scientific Research of Anhui Medical University, Hefei, China

**Keywords:** *IFIH1*, *TEF*, *TICAM1*, association study, single-nucleotide polymorphism, vitiligo

## Abstract

Forty-nine susceptible loci have been reported to be significantly associated with vitiligo by genome-wide association studies (GWASs) in European-derived whites. To date, some of these reported susceptibility loci have not yet been validated in the Chinese Han population. The purpose of this study was to examine whether the 16 reported susceptible loci in European-derived whites were associated with vitiligo in the Chinese Han population. Imputation was performed using our previous GWAS dataset by IMPUTE v2.2.2. The 16 imputed top single-nucleotide polymorphisms (SNPs) with suggestive signals, together with the reported SNPs, were genotyped in a total of 2581 patients and 2579 controls by the Sequenom MassARRAY system. PLINK 2.0 software was used to perform association analysis. The dbSNP database, HaploReg, and eQTL data were adopted to annotate the biological function of the SNPs. Finally, four SNPs from three loci were significantly associated with vitiligo, including rs3747517 (*P* = 1.29 × 10^–3^, OR = 0.87) in 2q24.2, rs4807000 (*P* = 7.78 × 10^–24^, OR = 0.66) and rs6510827 (*P* = 3.65 × 10^–5^, OR = 1.19) in 19p13.3, and rs4822024 (*P* = 6.37 × 10^–10^, OR = 0.67) in 22q13.2. According to the dbSNP database, rs3747517 is a missense variant of *IFIH1*, rs4807000 and rs6510827 are located in *TICAM1*, and rs4822024 is located 6 kb upstream of *TEF*. Further bioinformatics analysis by HaploReg and eQTL found that rs4807000, rs6510827, and rs4822024 are involved in regulating gene expression. Our study revealed the strong association of 2q24.2 (rs3747517), 19p13.3 (rs4807000, rs6510827), and 22q13.2 (rs4822024) with the risk of vitiligo in the Chinese Han population, which implicates common factors for vitiligo across different ethnicities, and helps expand the understanding of the genetic basis of this disease.

## Introduction

Vitiligo is an organ-specific autoimmune disease directed against melanocytes. It is characterized by whitish patches on the skin (Jin et al., 2019). The disease occurs in approximately 0.5–1% of the world population (Ezzedine et al., 2012). Most patients develop vitiligo before 40 years of age (Ezzedine et al., 2015). Vitiligo is also related to many other autoimmune diseases, in particular autoimmune thyroid disease, type 1 diabetes, and rheumatoid arthritis (Jin et al., 2019). The etiology of vitiligo remains elusive. There are several hypotheses, but an autoimmune etiology associated with specific genetic variants is still considered the leading theory (Iannella et al., 2016). It is believed that genetic components contribute to the onset of this disease (Spritz, 2012).

In 2010, we performed a genome-wide association study (GWAS) of vitiligo in the Chinese population, identifying 6p21.33, 6q27, and 10q22.3 as susceptible loci (Quan et al., 2010). Further two-stage replication studies identified three additional susceptibility loci at 10q22.1, 11q23.3, and 12q13.2 (Tang et al., 2013). From 2010 to 2018, the Spritz group carried out several large-scale GWASs in European-derived whites and discovered 49 novel susceptibility loci that contributed to vitiligo risk (Jin et al., 2010a,b, 2012, 2016; Ben et al., 2018). However, due to the genetic heterogeneity across different ethnicities, only some of these loci have been validated in the Chinese Han population, whereas others apparently have not been (Li et al., 2015; Xu et al., 2018; Tang et al., 2019). Of the 49 reported new susceptibility loci, we selected 16 single-nucleotide polymorphisms (SNPs) from 16 loci for validation after excluding those loci validated previously (Li et al., 2015; Xu et al., 2018; Tang et al., 2019). To investigate the association of the 16 loci with the risk of vitiligo, imputation of the 16 SNPs was conducted using our previous GWAS data on vitiligo, and the 16 imputed top SNPs together with the reported SNPs were genotyped in the Chinese Han population. Biological function annotation was performed to explain potential regulatory functions.

## Materials and Methods

### Study Subjects

All samples were recruited in collaboration with multiple hospitals in China. A total of 2581 unrelated patients (1335 men and 1226 women) and 2579 matched controls (1648 men and 931 women) were recruited for this study (Table 1). The patients were all of ethnic Chinese Han origin and diagnosed by at least two experienced dermatologists. Recruited controls who did not meet the following criteria were excluded: (a) healthy individual without vitiligo, any other autoimmune diseases, or systemic disorders; (b) Han nationality; (c) no family history of vitiligo (including of first-, second- and third-degree relatives). Written informed consent was obtained from all subjects before collecting their clinical information and blood samples. The Ethics Committee of Anhui Medical University approved this study protocol, and the study was carried out according to the ethical standards of the Declaration of Helsinki.

**TABLE 1:** Demographic details of the subjects.

	**Case**	**Control**
Total samples	2581	2579
Average age (age)(X ± SD)	28.53 ± 14.22	31.95 ± 15.05
Age arranged	1–85	1–91
Male	1355 (52.50%)	1648 (63.90%)
Female	1226 (47.50%)	931 (36.10%)

### SNP Selection for Replication

The genotype data of the 16 SNPs in adjacent upstream/downstream 250 kb regions were imputed from our previous vitiligo GWAS dataset (Quan et al., 2010). IMPUTE v2.2.2 was used to perform genotype imputation with the reference panel of the 1000 Genome Project phase 1 database (Howie et al., 2009). For quality control, SNPs that met the following criteria were included: (1) *P*-value of <10^–3^; (2) call rates >90%; (3) minor allele frequency (MAF) > 0.05 in controls; (4) *P*-value for Hardy–Weinberg equilibrium (*P*_HWE_) > 0.01 in controls. Then we selected the reported SNPs and the most significant SNPs in the16 loci (Table 2). Of those, the designs of the PCR primers for six SNPs failed. Finally, 26 SNPs within 16 loci were enrolled in the genotyping.

**TABLE 2:** Information about the 32 SNPs in the GWAS and 1000 genome data.

**Chr**	**SNP**	**Position^a^**	**Allele^b^**	**Minor Allele Frequency**		***P***	**MAF_CEU_**	**Origin**
				**Case**	**Control**			
1p36.23	rs301807	8424763	G/A	0.1605	0.1656	6.15 × 10^–1^	0.54	Reported
1p36.23	rs12745477	8204397	A/G	0.2432	0.2079	2.28 × 10^–3^	0.23	Imputed
2p16.3	rs6544997	47617366	A/G	0.3917	0.3733	1.65 × 10^–1^	0.50	Reported
2p16.3	rs72811506	47864584	T/A	0.1591	0.1314	4.92 × 10^–3^	0.01	Imputed
2q24.2	rs3747517	162272314	T/C	0.3357	0.3283	5.65 × 10^–1^	0.74	Reported
2q24.2	rs2300755	162027452	A/G	0.2781	0.3161	2.56 × 10^–3^	0.40	Imputed
3q13.33	rs59374417	119569567	A/C	0.2634	0.2563	5.51 × 10^–1^	0.11	Reported
3q13.33	rs16829716	119343558	A/G	0.0942	0.0686	5.65 × 10^–4^	0.09	Imputed
5q32	rs251464	149816671	G/C	0.2249	0.2092	1.63 × 10^–1^	0.25	Reported
5q32	rs9324634	149956876	G/A	0.0822	0.0630	6.26 × 10^–3^	0.12	Imputed
6p25.2	rs78521699	2908357	A/G	0.2122	0.2274	1.80 × 10^–1^	0.13	Reported
6p25.2	rs160669	2810535	C/T	0.2202	0.2551	4.03 × 10^–3^	0.82	Imputed
7p12.2	rs10250629	50151757	C/T	0.2454	0.2244	7.21 × 10^–2^	0.43	Reported
7p12.2	rs17633571	50132872	T/C	0.1842	0.1597	1.85 × 10^–2^	0.41	Imputed
8q24.22	rs2687812	132918810	A/T	0.3669	0.3810	2.85 × 10^–1^	0.53	Reported
8q24.22	rs1403486	132972870	A/G	0.2131	0.2440	8.43 × 10^–3^	0.62	Imputed
11q21	rs11021232	95587644	T/C	0.1031	0.0871	4.49 × 10^–2^	0.20	Reported
11q21	rs16922181	95556687	G/T	0.1598	0.1367	1.62 × 10^–2^	0.12	Imputed
13q14.11	rs35860234	42496070	T/G	0.1371	0.1046	2.36 × 10^–4^	0.29	Reported
13q14.11	rs146119164	42520465	T/A	0.1348	0.1015	1.52 × 10^–4^	0.18	Imputed
17q25.3	rs870355	78170115	G/A	0.4933	0.4924	9.46 × 10^–1^	0.41	Reported
17q25.3	rs749885	78270727	G/A	0.3335	0.2981	5.00 × 10^–3^	0.47	Imputed
18q21.31	rs10503019	57787145	G/A	0.0927	0.1014	2.80 × 10^–1^	0.23	Reported
18q21.31	rs7226959	57764310	T/C	0.1200	0.0913	7.58 × 10^–4^	0.30	Imputed
19p13.3	rs6510827	4830616	C/T	0.4382	0.4030	8.70 × 10^–3^	0.62	Reported
19p13.3	rs4807000	4831866	G/A	0.4396	0.4028	6.32 × 10^–3^	0.62	Imputed
19q13.3	rs2304206	49665614	G/A	0.1529	0.1808	1.07 × 10^–2^	0.26	Reported
19q13.3	rs12986313	49455384	C/A	0.2168	0.2474	1.16 × 10^–2^	0.40	Imputed
21q22.11	rs2833607	32008727	G/A	0.2672	0.2443	5.25 × 10^–2^	0.23	Reported
21q22.11	rs9305469	31779113	T/G	0.1584	0.1310	4.19 × 10^–3^	0.85	Imputed
22q13.2	rs4822024	41361643	G/A	0.0880	0.1031	6.62 × 10^–2^	0.24	Reported
22q13.2	rs5758361	41456728	A/C	0.4302	0.3860	1.50 × 10^–3^	0.00	Imputed

*Chr, chromosome; SNP, single-nucleotide polymorphism; MAF_*CEU*_, minor allele frequencies of SNPs in European population from 1000 Genome Project phase 1 database. ^*a*^Positions are based on human genome version 38 (hg38). ^*b*^Minor allele/major allele.*

### DNA Extraction and Genotyping

Genomic DNA was extracted from blood samples using FlexiGene DNA kits (QIAGEN, Hilden, Germany) according to the manufacturer’s protocol. After the concentration and purity were measured using a spectrophotometer, the 26 SNPs were genotyped using the Sequenom MassARRAY platform (Sequenom, San Diego, CA, United States). The procedures used for genotyping were presented in a previous study (Cai et al., 2019).

### Statistical Analysis

PLINK 2.0 software was used to calculate the allele frequency, *P*-values, odds ratios (OR), and Hardy–Weinberg equilibriums (HWE) for each SNP. The association of vitiligo with the SNPs was analyzed by comparing the MAF between patients and controls. The statistical power was calculated using Power and Sample Size Calculation software using either the sample size, the MAFs observed in the Chinese controls, and the ORs previously reported for European-derived whites, or the ORs in our GWAS and the imputed results for the Chinese Han when they were not provided for the white European population. For quality control, SNPs with a call rate < 90% and *P*_HWE_ < 0.01 were excluded. A *P*-value after Bonferroni corrections of less than 3.57 × 10^–3^ (0.05/14) was regarded as statistically significant.

### Bioinformatics Analysis

Several bioinformatics tools were utilized in this study. The Single Nucleotide Polymorphism database (dbSNP) was used for gene mapping.^1^ HaploReg4.1^2^ was adopted to select the strongly linked SNPs and evaluate the potential biological significance for targeted SNPs. In addition, expression quantitative trait loci (eQTL) study data based on the GTEx database^3^ were adopted (GTEx Consortium, 2013).

## Results

### Association Between SNPs and Vitiligo

In this study, 26 selected SNPs from 16 loci were genotyped in 2581 vitiligo patients and 2579 healthy controls. Twelve SNPs were eliminated for not meeting the inclusion criteria (call rate < 90%, *P*_HWE_ < 0.01), leaving 14 SNPs for analysis. Four SNPs showed significant associations with vitiligo in the independent Chinese Han cohort, including rs3747517 (*P* = 1.29 × 10^–3^, OR = 0.87, 95% CI: 0.80–0.95) at 2q24.2, rs4807000 (*P* = 7.78 × 10^–24^, OR = 0.66, 95% CI: 0.61–0.71) and rs6510827 (*P* = 3.65 × 10^–5^, OR = 1.19, 95% CI: 1.09–1.29) at 19p13.3, and rs4822024 (*P* = 6.37 × 10^–10^, OR = 0.67, 95% CI: 0.58–0.76) at 22q13.2 (Table 3). However, no evidence of a significant association was observed for any other SNPs.

**TABLE 3:** Summary of association results of 14 SNPs in 11 loci/genes between cases and controls.

**Chr**	**SNP**	**Position^a^**	**Allele^b^**	**Minor Allele Frequency**		***P*-value^c^**	**OR (95% CI)**	***P*_HWE_**	**Call-Rate**	**Power**	**Allele_ceu_**	**OR_ceu_**	***P*-value_ceu_**
				**Case**	**Control**								
1p36.23	rs12745477	8204397	A/G	0.2228	0.2172	4.95 × 10^–1^	1.03 (0.94–1.14)	2.35 × 10^–1^	0.96	0.569*	–	–	–
2p16.3	rs6544997	47617366	A/G	0.3786	0.3971	5.76 × 10^–2^	0.92 (0.85–1.00)	1.20 × 10^–1^	0.97	0.062*	A/G	*	6.61 × 10^–6^
**2q24.2**	**rs3747517**	**162272314**	**T/C**	**0.3298**	**0.3615**	**1.29 × 10**^–^**^3^**	**0.87 (0.80**–**0.95)**	**1.98 × 10**^–^**^2^**	**0.91**	**0.924**	**T/C**	**0.78**	**1.65 × 10^–13^**
2q24.2	rs2300755	162027452	A/G	0.3114	0.3038	4.21 × 10^–1^	1.04 (0.95–1.13)	4.07 × 10^–1^	0.94	0.555*	–	–	–
3q13.33	rs59374417	119569567	A/C	0.2644	0.2567	3.80 × 10^–1^	1.04 (0.95–1.14)	2.67 × 10^–1^	0.97	0.968	A/C	1.34	5.41 × 10^–1^
6p25.2	rs160669	2810535	C/T	0.2446	0.2315	1.32 × 10^–1^	1.08 (0.98–1.18)	2.25 × 10^–1^	0.93	0.529*	–	–	–
7p12.2	rs10250629	50151757	C/T	0.2354	0.2252	2.34 × 10^–1^	1.06 (0.96–1.17)	9.07 × 10^–1^	0.94	0.171	C/T	0.88	2.11 × 10^–7^
11q21	rs11021232	95587644	T/C	0.09605	0.09108	4.04 × 10^–1^	1.06 (0.92–1.22)	8.04 × 10^–1^	0.93	0.613	C/T	1.34	2.10 × 10^–23^
11q21	rs16922181	95556687	G/T	0.1454	0.1373	2.52 × 10^–1^	1.07 (0.95–1.20)	8.62 × 10^–1^	0.94	0.282*	–	–	–
18q21.31	rs10503019	57787145	G/A	0.09699	0.1017	4.43 × 10^–1^	0.95 (0.83–1.09)	8.23 × 10^–1^	0.94	0.053*	A/G	*	2.7 5 × 10^–6^
**19p13.3**	**rs6510827**	**4830616**	**C/T**	**0.4166**	**0.3755**	**3.65 × 10**^–^**^5^**	**1.19 (1.09**–**1.29)**	**1.08 × 10**^–^**^1^**	**0.94**	**0.556**	**C/T**	**1.19**	**6.37 × 10**^–^**^1^**
**19p13.3**	**rs4807000**	**4831866**	**G/A**	**0.4236**	**0.5273**	**7.78 × 10**^–^**^24^**	**0.66 (0.61**–**0.71)**	**7.16 × 10**^–^**^2^**	**0.91**	0.415*	–	–	–
21q22.11	rs9305469	31779113	T/G	0.1468	0.1448	7.73 × 10^–1^	1.02 (0.91–1.14)	8.70 × 10^–1^	0.96	0.501*	–	–	–
**22q13.2**	**rs4822024**	**41361643**	**G/A**	**0.08973**	**0.129**	**6.37 × 10**^–^**^10^**	**0.67 (0.58**–**0.76)**	**2.24 × 10**^–^**^2^**	**0.94**	**0.588**	**G/A**	**0.78**	**2 × 10**^–^**^1^**

Chr, chromosome; SNP, single-nucleotide polymorphism; OR, odds ratio; CI, confidence interval; HWE, Hardy–Weinberg equilibrium. ^*a*^Positions are based on human genome version 38 (hg38). ^*b*^Minor allele/major allele. ^*c*^*P-*values of less than 3.57 × 10^–3^ (0.05/14) were considered to be statistically significant (with Bonferroni corrections); Allele_*ceu*_, OR_*ceu*_, *P*-value_*ceu*_ are reported by vitiligo GWASs in European-derived whites; “–” Imputed SNPs. *The odds ratio is unavailable from European GWASs and power is calculated by using the MAFs observed in Chinese controls and the ORs previously reported for Chinese. The bold values implicate positive results.

### Functional Annotation via Bioinformatics Analysis

The positive vitiligo SNPs rs4807000 and rs6510827 are in the strong linkage disequilibrium (LD) region (*r*^2^ = 0.99) of 19p13.3 (Supplementary Figure 1), separately in 140 bp upstream and intronic region of *TICAM1.* According to Haploreg v4.1, these two SNPs are in the area of enhancer histone H3K4mel in primary melanocyte cells and primary keratinocyte cells. The eQTL data extracted from GTEx show that they are associated with the expression of *TICAM1* in whole blood (*P* = 1.70 × 10^–36^; *P* = 2.13 × 10^–36^, respectively). Moreover, rs4807000 is in the binding site of the NF-κB transcription factor from the ENCODE project.

Rs4822024 is located 1.5 kb downstream of *ZC3H76*, 6 kb upstream of *thyrotroph embryonic factor* (*TEF*), and 15 kb upstream of *RNU6-495P* in 22q13.2 (Supplementary Figure 2). This SNP shows a significant QTL effect on the expression level of *TEF* in the blood (*P* = 1.45 × 10^–14^) (GTEx Consortium, 2015) and is in the region of enhancer histone H3K4me1 in primary melanocyte cells according to Haploreg v4.1. Furthermore, the SNP also contains enrichment enhancer and promoter histone marks in epithelial cells, which suggests a transcriptional regulation function.

Gene mapping showed that rs3747517 is a missense variant in the *interferon-induced helicase C domain 1 (IFIH1)* gene by dbSNP (Supplementary Figure 3). As a vitiligo-protective variant, the functional analysis of rs3747517 has been addressed previously (Jin et al., 2017).

## Discussion

Vitiligo is a common depigmenting skin disorder influenced by genetic and environmental factors. Our team conducted a series of genetic studies of vitiligo in the Chinese Han population. A GWAS of vitiligo was carried out in 2010. The 34 most promising SNPs in the MHC region were replicated, and one new risk locus was identified at 6q27, which contains three genes, *RNASET2*, *FGFR1OP*, and *CCR6* (Quan et al., 2010). In 2013, the previous vitiligo GWAS was extended with a two-stage replication study, and 50 SNPs at 44 loci showing a suggestive association (*P*_initial_ < 1 × 10^–4^ in GWAS) were selected for replication testing, which identified three susceptibility loci (12q13.2, 11q23.3, and 10q22.1) for vitiligo (Tang et al., 2013). In 2015, 12q13.2 was verified to be significantly associated with vitiligo accompanying immune-related diseases by testing 10 immune susceptibility SNPs (Li et al., 2015). In 2017, two newly identified SNPs (rs613791 and rs523604) showed independent signals in the associated locus 11q23.3 for vitiligo (Zhao et al., 2017). In 2019, 3q29 was demonstrated to be associated with vitiligo after genotyping 14 reported loci identified from a meta-analysis of GWAS in European-derived whites (Tang et al., 2019). In this study, 16 reported susceptible loci (1 × 10^–3^ > all *P_initial_* > 1 × 10^–4^ in our GWAS, located in non-MHC regions) in European-derived whites by GWAS were first tested to be associated with vitiligo in the Chinese Han population (Table 2), which revealed the strong association of 2q24.2 (rs3747517), 19p13.3 (rs4807000 and rs6510827), and 22q13.2 (rs4822024) with vitiligo.

SNP rs3747517 in 2q24.2 is a missense variant of *IFIH1* encoding the IFIH1 protein, which can activate innate immune reaction to bind to damage-associated molecular patterns (Looney et al., 2015). Dysfunction of *IFIH* impairs the activation of downstream innate immune responses (Jin et al., 2017). SNPs rs4807000 and rs6510827 in 19p13.3 are located separately in 140 bp upstream and the intron of *TICAM1* with significant LD (*r*^2^ = 0.99). *TICAM1* regulates the innate immune reaction to viruses by inducing pattern recognition receptor–mediated IFN production (Seya et al., 2009). SNP rs4822024 in 22q13.2 is located 6 kb upstream of *TEF*, which encodes the TEF protein, and is associated with the expression level of TEF in blood (GTEx Consortium, 2015). TEF is a member of the proline and acidic amino-acid-rich basic leucine zipper (PAR bZip) transcription factor family, which plays a pivotal role in regulating circadian rhythm. The above loci may be involved in the pathogenesis of vitiligo through immune or other mechanisms.

The current findings revealed four SNPs in three loci that contribute to vitiligo susceptibility in Chinese Han individuals. However, we were unable to obtain evidence of an association for the other 10 SNPs. Aside from these 14 SNPs, 6 SNPs failed in the assay design, and 12 SNPs were eliminated for not meeting the quality-control criteria. The MAFs of these SNPs exhibited some difference between the Chinese Han (CHB) and European (CEU) cohorts (Table 2), and the correlation identified in the European population may not be well present in this study. Power analysis showed that the sample of 2581 patients and 2579 controls provided insufficient statistical power (<80%) for these negative SNPs except for rs59374417 in 3q13.33, which was not sufficient for detecting an association (Table 3), so larger sample sizes are needed in future studies. In addition, the difference in association evidence for rs59374417 between the Chinese Han and European-derived whites may implicate a genetic heterogeneity of vitiligo susceptibility between ethnic populations.

In this study, we tested the association of 16 susceptible SNPs for vitiligo identified in European-derived whites and the respective top imputed SNPs of candidate regions in a Chinese Han population. The negative results only indicated that these SNPs, not their LD region, were not related to vitiligo in the Chinese Han population. The differences in LD structure around the causal variants might result in distinct observations of associations in different populations. A more in-depth comparison of the LD structures in the candidate regions in Chinese Han versus European populations is necessary, either by targeted sequencing or fine genotyping in future studies.

In conclusion, the strong association of 2q24.2 (rs3747517), 19p13.3 (rs4807000 and rs6510827), and 22q13.2 (rs4822024) with the risk of vitiligo was demonstrated in a Chinese Han population, which implicates common factors for vitiligo across different ethnicities, and helps expand the understanding of the genetic basis of this disease.

## Data Availability Statement

All datasets generated for this study are included in the article/Supplementary Material.

## Ethics Statement

The studies involving human participants were reviewed and approved by the Ethics Committee of the Anhui Medical University. Written informed consent to participate in this study was provided by the participants.

## Author Contributions

F-LX designed and supervised the study. LC wrote and revised the manuscript. X-YC helped to analyze the data. X-FT helped to select the SNPs. BL and HC enrolled the patients. LC, JiZ, MW-W, and JuZ conducted the experiments. X-DZ processed the data and performed statistical analysis. F-SZ and PL helped with genotyping. All authors contributed to the article and approved the submitted version.

## Conflict of Interest

The authors declare that the research was conducted in the absence of any commercial or financial relationships that could be construed as a potential conflict of interest.

## Footnotes

1 http://www.ncbi.nlm.nih.gov/snp

2 https://pubs.broadinstitute.org/mammals/haploreg/haploreg.php

3 http://www.gtexportal.org/home/
