# Supplementary figures and images for: Validation of Susceptibility Loci for Vitiligo Identified by GWAS in the Chinese Han Population
Source: Front Genet. 2020 Dec 3;11:542275. doi: 10.3389/fgene.2020.542275 (PMC7744663; doi:10.3389/fgene.2020.542275)

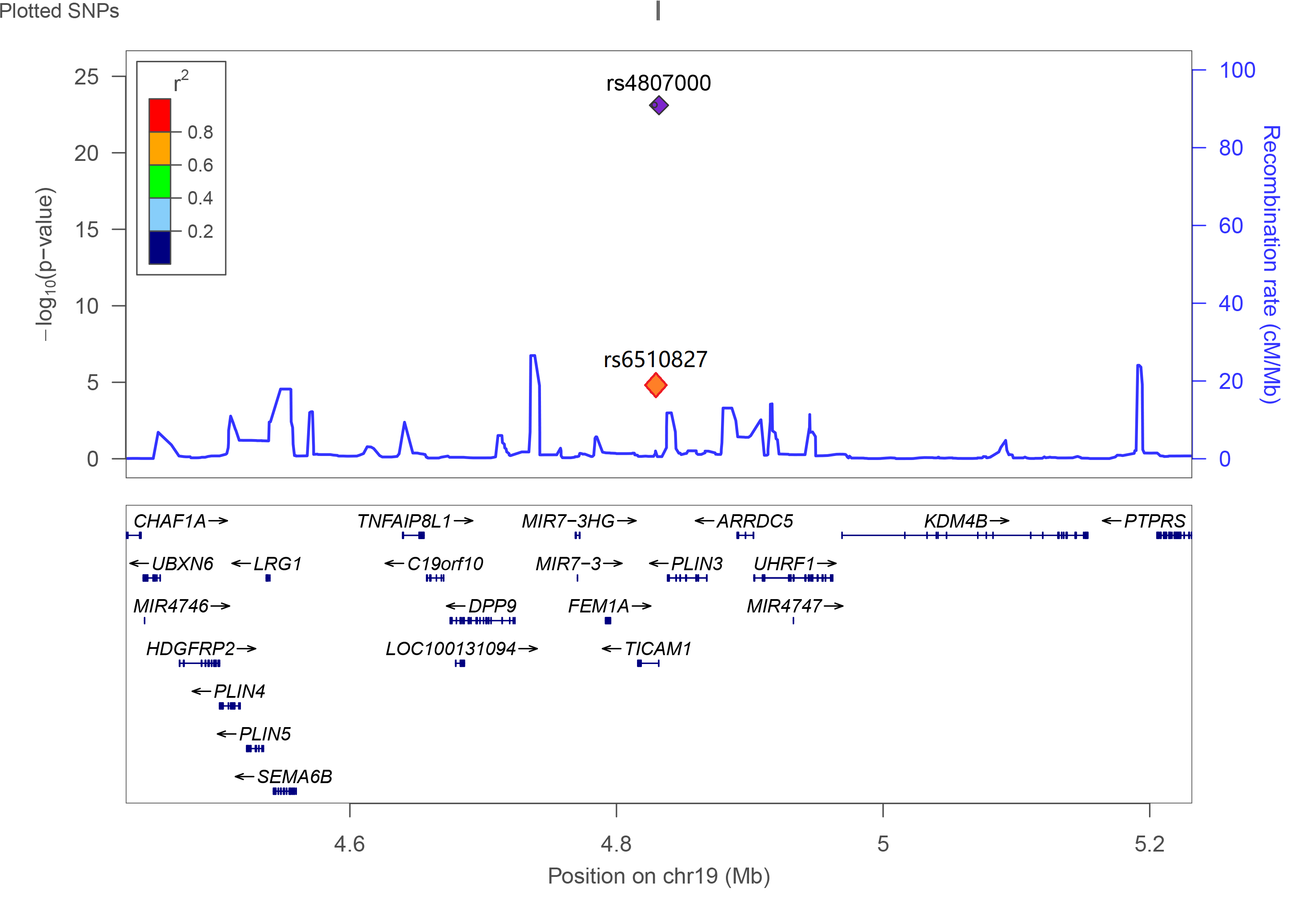

Supplement: Supplementary file 1 [file Image_1.TIF]

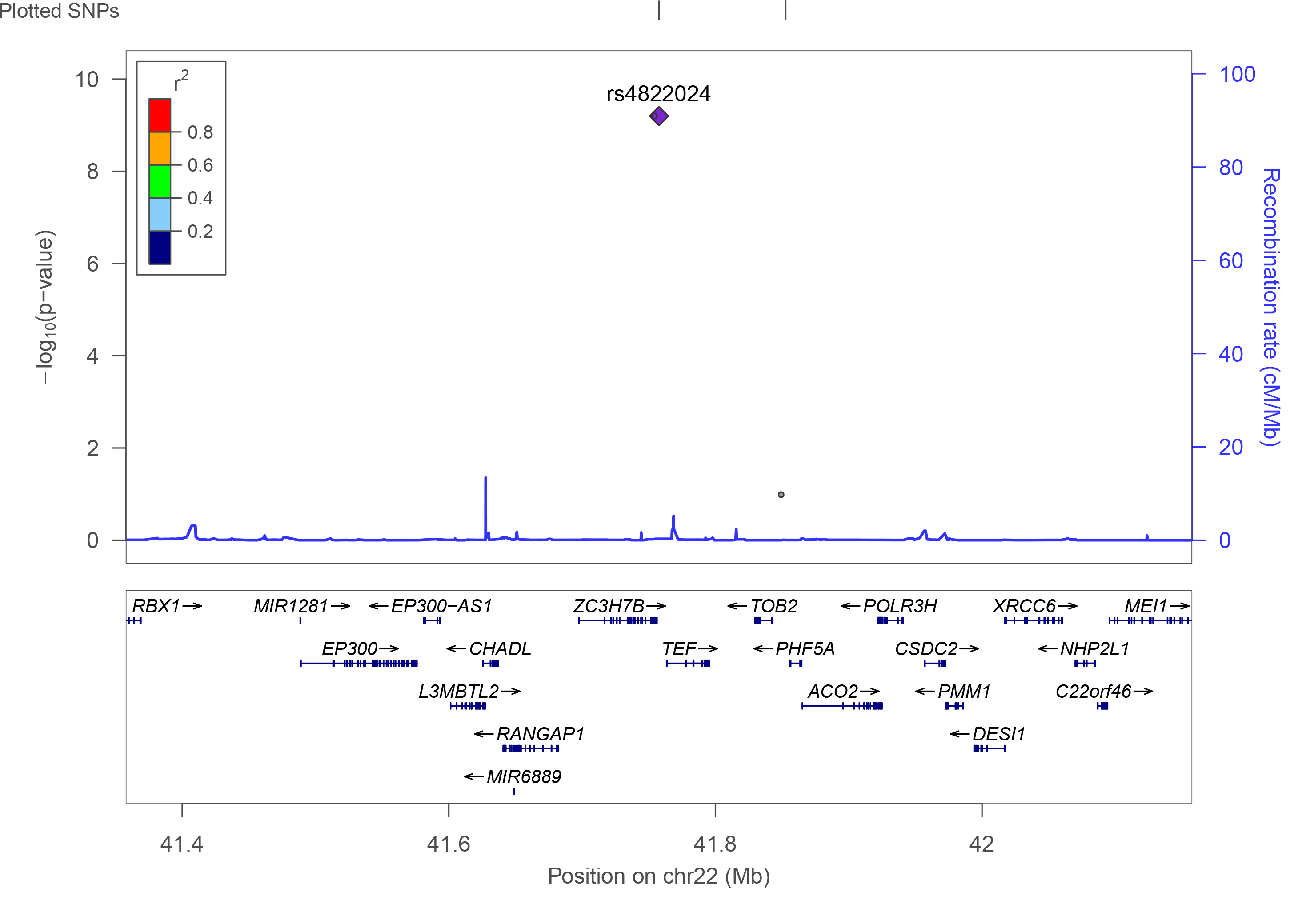

Supplement: Supplementary file 2 [file Image_2.TIF]

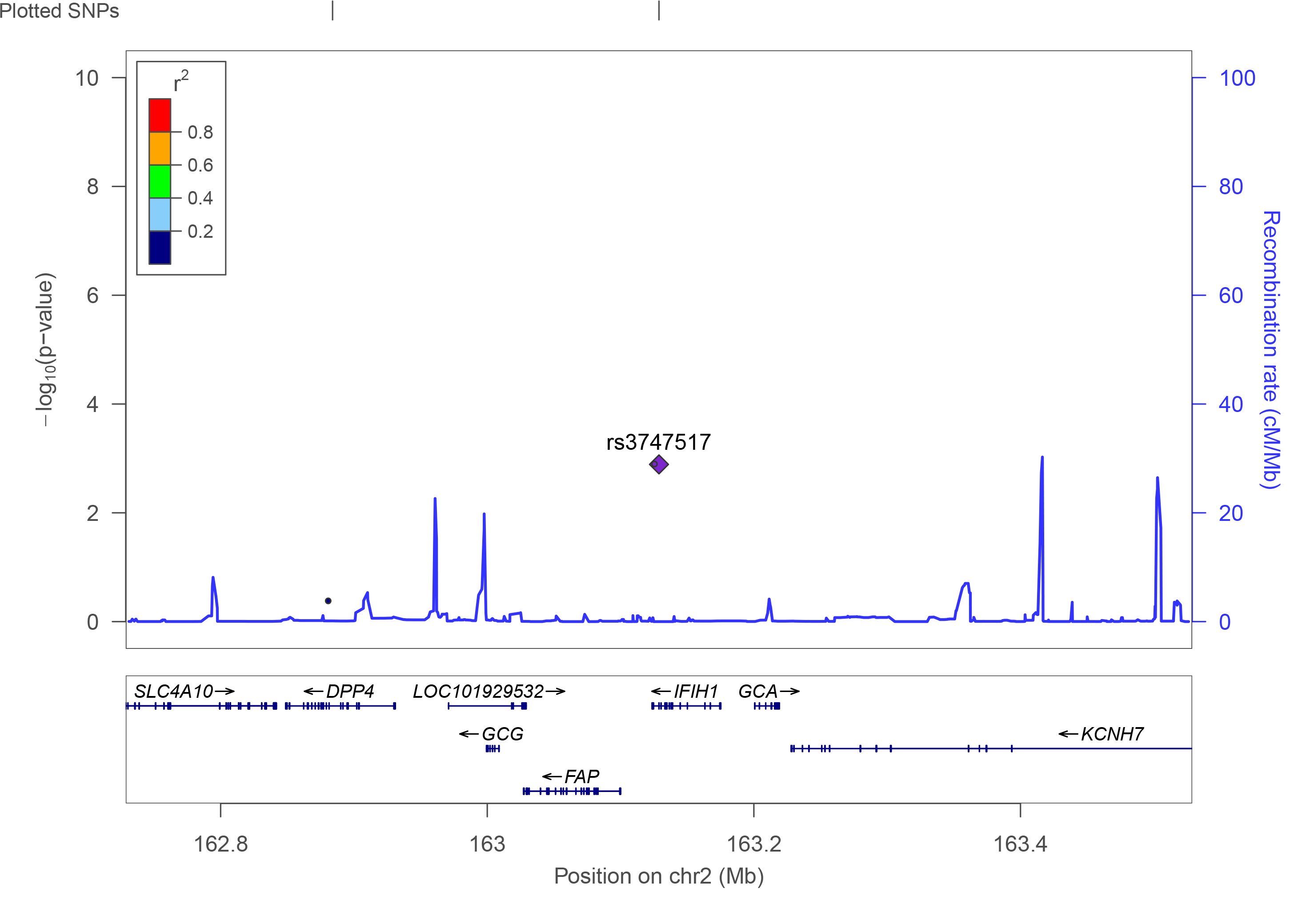

Supplement: Supplementary file 3 [file Image_3.TIF]
